# Natural ventilation cooling effectiveness classification for building design addressing climate characteristics

**DOI:** 10.1038/s41598-024-66684-9

**Published:** 2024-07-13

**Authors:** Wenjing Li, Xinhui Xu, Jiawei Yao, Qingchang Chen, Zhuoyang Sun, Mehdi Makvandi, Philip F. Yuan

**Affiliations:** 1 College of Architecture and Urban Planning, Tongji University, Shanghai, 200092 People’s Republic of China; 2 School of Architecture, Shanghai Institute of Technology, Shanghai, 200235 People’s Republic of China; 3 Key Laboratory of Ecology and Energy-Saving Study of Dense Habitat, Ministry of Education, Shanghai, 200092 People’s Republic of China; 4 College of Civil Engineering, Zhejiang University of Technology, Hangzhou, 310058 People’s Republic of China

**Keywords:** Natural ventilation potential, Energy efficient building design, Normative model, Critical parameters, Climate characteristics, Climate-change policy, Energy and society

## Abstract

The evaluation of natural ventilation potential for effective sustainable options and innovative green building design strategies is of great interest to architects, researchers and governments. From a retrospective review, we found that the potential evaluation of natural ventilation (NV) cooling effectiveness in the same category based on similar meteorological uncertainty, research objectives and objects showed significant differences. Uncertainties added and uncertainty propagation (both model form uncertainties and parameter uncertainties) could result in large discrepancies between simulation outcomes and real scenarios, especially in the design performance modeling (DPM) phase. In this conceptual design stage, a few parameters are available and therefore decisive. It is necessary to review and identify the key performance indicators and explore the extent to which deviations are caused by inconsistencies or biases in model information. As a basis for more concrete research, we propose statistical tests based on quantitative evaluations to explore the rule of natural ventilation potential volatility and identify whether there is a significant potential improvement resulting from the critical parameter enhancement with the optimal relationship. The showcase is applied in China, where there has been a significant amount of criticism regarding the current building climate zoning due to the perceived coarseness of the system and where there has been an active exploration into the possibility of redefining building climate zoning with a view toward improving its accuracy and effectiveness.

## Introduction

### Natural ventilation potential in the preliminary design stage

As excessive carbon emissions contribute to climatic variation^1,2^, consensus has been reached in leading economies worldwide to reduce energy consumption, which in buildings as direct services currently accounts for approximately 40% of the total social energy consumption in Europe^3,4^. For countries or regions with rapid urbanization, such as China, the account is 31%^5^. The tension between resource demand and thermally comfortable indoor environment maintenance is mounting. Natural ventilation (NV) has been recognized to deliver substantial cooling-energy reductions of 40–50% in some major urban areas in Europe and North America^6–9^ and 20–40% in the Asian continent^10–12^.

Mechanically controlled indoor environments tend to be static and homogeneous^13^. Critics regard it as an expensive solution that results in thermal monotony. Only a 59% mean building satisfaction rate was found through a large survey of more than 200 commercial building occupants, which is considerably lower than the minimum requirement of thermal comfort in ASHRAE standard 55^14^. Questioning around this static environment is sharpened by the coronavirus (COVID-19) global pandemic with the evidence that some circumstances, for example, being indoor environments where ventilation with inadequate outside air, will increase the infection risk. Not surprisingly, static environments are giving way to more dynamic environments, in which wider ranges of indoor temperature are preferred and NV is desired^15,16^. Generally, in the context of COVID-19 causing severe damage to the local economy, companies do not have the resources to immediately replace advanced mechanical ventilation equipment to prepare for the next round of epidemic impact and protect the personal health and work efficiency of employees after the lifting of control measures. However, some of them immediately require employees to return to their positions to ensure normal operations. In this case, natural ventilation is one of the mainstream choices. It has a relatively low dependence on equipment and can return control of the indoor environment to individuals^17^.

Against this backdrop, resilient built environment generation, which includes building design to maximize occupant satisfaction and satisfaction by defending while harmonizing local conditions, is identified as one of the paramount research challenges and opportunities. For Architecture, the methodology is evolving from traditional composition to evidence-based design proposal or performance-oriented and performance-aware morphology generation. As a complexity philosophy is forming, it tends to treat this morphology generation as a complex adaptive organization best surviving and developing in a dynamic environment^18^. Natural ventilated buildings, generally accepted to be subject to a special form, are of great interest to architects as an innovative design option^19,20^. Assisting in design development processes, the metrics quantify how well indoor spaces make use of natural ventilation cooling capacity.

Researchers^7^ are concentrating on identifying the key parameters of NV and proposing dynamic metrics responsive to various design options, i.e., assisting in design development processes and quantifying how well special forms make use of NV capacity. However, there is still a range of deficiencies that need to be overcome. For instance, during the architectural design process, first, the quantity finiteness of core parameters is argued to be necessary. In the conceptual design stage, only a few parameters are available for design performance modeling (DPM). Ultimately, to compare numerous building massing designs—particularly those based on the evolutionary algorithm—it is imperative to obtain rapid feedback from multiple analyses. In this sense, a normative energy performance calculator (EPC) is recommended in this paper, which has been proven to be a nonsimulation-based adaptation of the ASHRAE 90.1 calculation approach^21^. This EPC normative method executes the calculations specified in ISO 52016^22^.

### Interpretation of design-based natural ventilation potential

**Figure 1:**
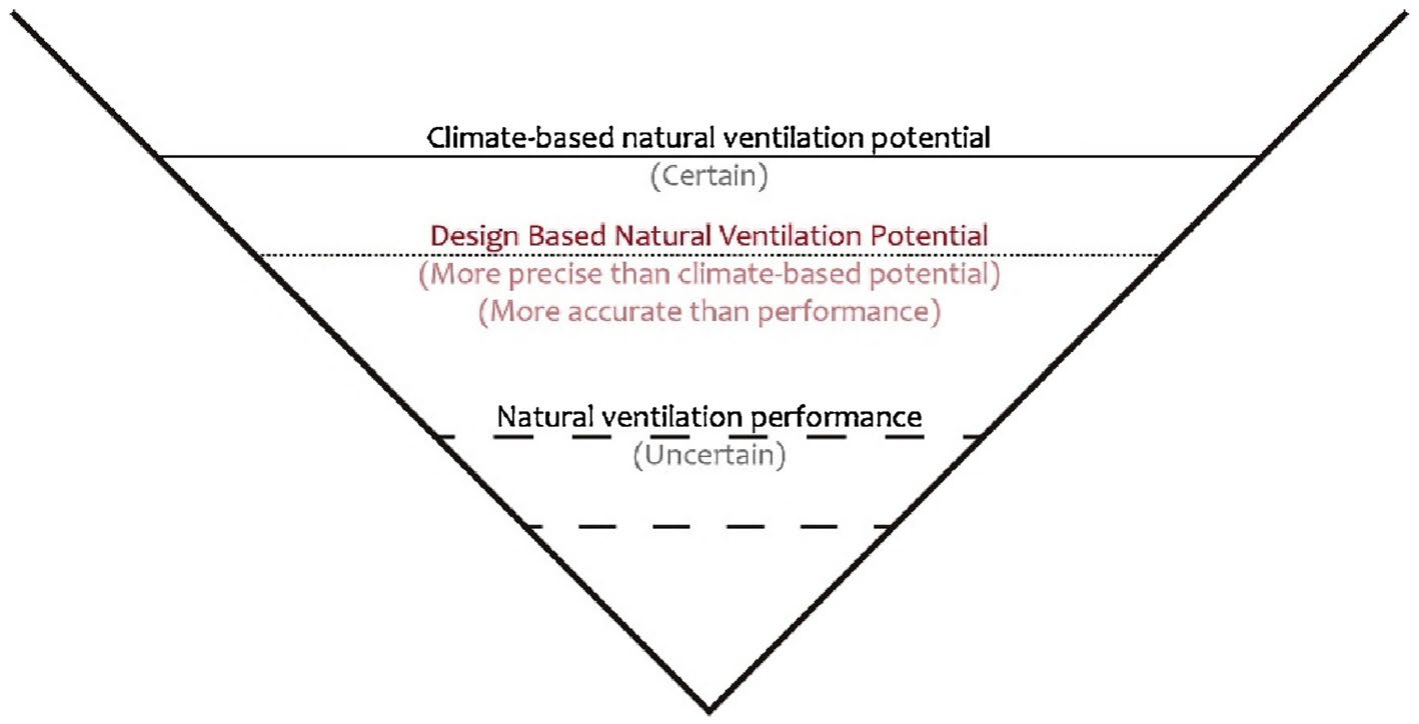
Diagram of the natural ventilation cooling potential^29^.

The air regulation of naturally ventilated buildings depends on a combination of natural ventilation systems or pure natural ventilation systems. Currently, the most widely discussed are commercial buildings with self-controlled exterior windows and cooling capabilities. Exterior windows refer to the windows directly facing the outside of the building and their variations, such as Godfried Augenbroe's line-shaped opening integrated in the window frame^23^. These buildings, based on the whole-air conditioning system, use self-controlled exterior windows to replace part or all of the mechanical ventilation with natural ventilation as the cooling source, thereby achieving energy-saving purposes without reducing human thermal comfort. The ASHRAE Standard 55–2020 in the United States has established targeted indoor thermal comfort standards for such naturally ventilated buildings^24^. This project adopts this definition of naturally ventilated buildings. The maximum value of evaluation indicators that can be introduced into target buildings represents the NVP, which represents the performance of naturally ventilated buildings at each design stage. During the primary design stage, such as the schematic design or predesign analysis phase^25^, as the building design process progresses, the model information becomes more abundant and the evaluation methods are refined, the NVP values show a decreasing trend (Fig. 1). Conventional criteria restricting the NV potential interval are as follows: (1) Annual hours when acceptable indoor conditions could be provided by NV, e.g., NV hours (various metrics including satisfied natural ventilation hour (SNVH)^10^). (2) Building energy performance indicators normally denote cooling effectiveness (various metrics, including climate cooling potential (CCP)^26^). (3) Air change rate (ACH) (related indicators include airflow rates and air speeds and pressure difference Pascal hours (PDPH)^11,27,28^). Our previous study reveals that the rate of NV financial saving effectiveness (kdollar/m^2^) is directly related to cooling load reduction (i.e., cooling effectiveness). The main difference reflects the value of the COP of the HAVC (excluding pumps and fans) and the average price of electricity in a given location. In this study, the criteria NV hours and cooling effectiveness are employed.

**Figure 2:**
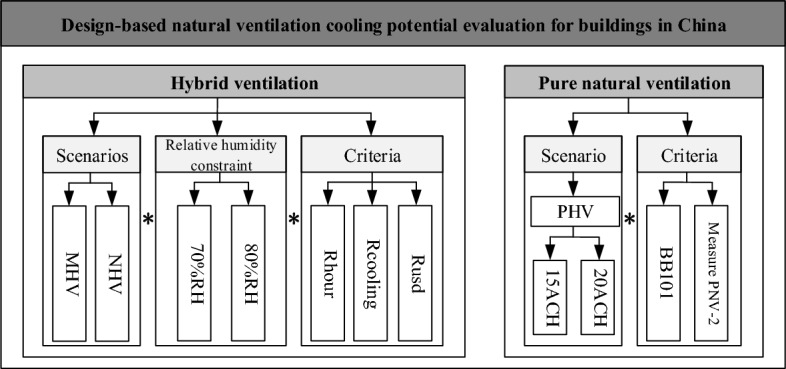
The mainframe structure of NV cooling potential evaluation^30^.

Scenarios investigated in previous studies can be summarized as hybrid ventilation (HV) and pure natural ventilation (PNV) (Fig. 2). In the HV situation, the needs in terms of cooling and fresh air supply are partially fulfilled by natural means, with the realistic option of controllable ventilation openings and cooling system operation for buildings. When the indoor temperature is greater than the setpoint, a cooling system is operated to maintain the temperature. Outside these hours, if the mechanical system supplies cooling with fresh air, this alternative is defined as the MHV scenario (mechanical hybrid ventilation). If the mechanical system supplies cooling and fresh air continues to supply ambient air, it is defined as the NHV scenario (natural hybrid ventilation). PNV is defined as all possible demands above that are provided by NV. A lack of mechanical alternatives will result in temporary thermal discomfort, but PNV situation setting is necessary for developing regions, where a full air-conditioned indoor environment that matches resident habits or markets has not formed or where a tradition and acceptance of full naturally ventilated buildings has been held. In previous studies, it has been proven that (1) PNV is partly available in certain regions and seasons in China, even though it is highly recommended. (2) The additional energy savings achieved by exhaust fans supplying fresh air outside of NV hours in a calendar year are not significant in China. (3) In all scenarios, the addition of night ventilation strategies and the relative humidity (RH) cutoff for allowable ventilation should be considered. Hence, in this study, the NHV scenario is chosen to represent the NV potential in the primary design phase. In addition, 70% RH is employed according to GB 50,189–2015.

### General uncertainties added and uncertainty propagation

Based on system scales, uncertainties in building performance assessments can be divided into five categories, i.e., the uncertainty of meteorological scale, urban scale, building scale, system scale and operation scale^14^. Meteorological uncertainty is driven by weather fluctuation and possible climate changes in the thermal climate zone. A significant deviation of weather data for estimation from the realistic scenario could contribute to a large discrepancy between computed results and objective existence^31^. To address this uncertainty, Lujian Bai et al.^32^ determined the jump phenomena of climate using the moving t test method and reassigned cities to new thermal climate zones based on building energy estimation. In the primary design stage, i.e., DPM progress, the urban uncertainty influenced by the building microclimate environment, e.g., the urban heat island effect, is difficult to quantify for a single template building. Of course, in an automated design process, a generative adversarial network (GAN) could be applied in a surrogate model as an accelerator for environmental performance-driven urban design. This progress is a so-called “black box”, which means that it cannot be traced and explained^33^. This is a more broad-brush, macrolevel method that is appropriate for strategic, overall planning, or design decision-making. Hence, in this research, urban scale uncertainty was not considered. Building uncertainties arise mainly from the possible variation of material properties and approximation of physical processes in the building simulation. Nari Yoon^34^ proposes two new metrics (NV cooling effectiveness and climate potential utilization ratio) for dynamic response to various design options in both steady and transient states, allowing consideration of thermal mass. Cristina Baglivo studied how envelope design optimization impacts a building's thermal behavior in a certain warm climate^35^. System and operation uncertainties, in the primary stage, mainly originated from the variability of HVAC equipment efficiency, system operation controls, gas, and electricity consumption and possibly could be related to the prediction of occupant behaviors^36^.

Because of the significant simplification of the DPM, the influence of some specific available parameters on potential evaluation will be increased. This makes it essential to be aware of modeling input variables. The variables can be divided into four categories: building basic geometries, envelope properties, internal gains, and HVAC systems^21^. For the NV potential estimation, it was validated that specific HVAC system type information plays a role only for periods when the investigations are translated into absolute monetary savings, as most actual HVAC systems fall within a narrow range of COP values and auxiliary electricity use. In the previous parametric analysis, it is typically believed that envelope properties, which are prescribed by native energy conservation regulation, have a major effect on NV cooling energy consumption, as important as indoor temperature set point optimization.

In this sense, this paper aims to explore the extent to which deviations are caused by inconsistencies or biases in model information in the DPM phase. Statistical tests based on quantitative evaluations are proposed to explore the rule of natural ventilation potential volatility and identify whether there is a significant potential improvement resulting from critical parameter enhancement with the optimal relationship. The potential variation curves caused by different parameters are compared, especially the correlations between the building envelope, thermal mass requirements, and main operating schedule of the temperature control system, and a reliable value range is proposed. The comparative experiment is applied to five climate zones of China as a showcase.

### Dissonance metrics for natural ventilation potential in China

Examples of China are illustrated to explain the dissonance discussed above. Comparable previous studies of NV hours are displayed in Table 1 It is shown that the performance of the same object city shows significant differences, which even affect the potential ranking of climate zones (papers published after 2010 and focus on nonspecific building ventilation are selected). In other words, even if the performance is only used for horizontal comparison (i.e., comparative study), the results will have deviations.

**Table 1:** Previous studies of design-based natural ventilation cooling potential evaluation for buildings in China^40,41^.

	**Scenarios & Criteria**			**Climate & Configuration data**		**Simulator/Core methodology**	**Evaluation purpose /Highlight**	**Climate zone (Cities)**	**Annual NV hours (hr)**
^37^	1. ASHRAE Handbook-Fundamentals-2009 2. ASHRAE 55–2009			**Climate data**	Chinese Standard Weather Data (CSWD)	1. EnergyPlus 2. Fundamental heat balance principle	1. To prove the impact of ambient air pollution on NV potential 2. 8–78% of cooling energy usage can potentially be reduced by NV	5 (76)	Harbin (2091) Beijing (2771) Shanghai (2365) Kunming (6047) Guangzhou (2898)
	**Outdoor threshold**	**Outdoor dry-bulb temperature and Humidity**	1. Adaptive thermal comfort model 2. > 12.8℃	**Configuration data**	GB 50,189–2015: Chinese Design Standard for Energy Efficiency of Public Buildings				
		**Airflow**	None	**Ventilation strategy**	1. Mechanical ventilation 2. Hybrid ventilation				
^38^	**Scenarios & Criteria**			**Climate & Configuration data**		**Simulator/Core Methodology**	**Evaluation purpose/Highlight**	**Climate zone (Cities)**	**Annual NV hours** **(hr)**
	1. ASHRAE Standard 169–2006 2. ASHRAE Standard 90.1–2007 3. ASHRAE Standard 55–2009 4. ASHRAE Handbook-Fundamentals-2009			**Climate data**	1. Typical Meteorological Year 3 (TMY3) 2. International Weather for Energy Calculations (IWEC) 3. CSWD	1. EnergyPlus 2. Fundamental heat balance principle	To estimate the NV potential of 1854 locations around the world and calculate energy saving potentials of the world’s 60 largest cities	5 (1854)	Harbin (2356) Beijing (2651) Shanghai (2302) Kunming (5566) Guangzhou (2434)
	**Outdoor threshold**	**Outdoor dry-bulb temperature and Humidity**	Adaptive thermal comfort model	**Configuration data**	1. U.S. Department of Energy (DOE) commercial reference building database 2. International Energy Conservation Code (IECC) 2009				
		**Airflow**	ASHRAE Standard 55	**Ventilation strategy**	1. Mechanical ventilation 2. Hybrid ventilation				
^39^	**Scenarios & Criteria**			**Climate & Configuration data**		**Simulator/Core Methodology**	**Evaluation purpose/Highlight**	**Climate zone (Cities)**	**Annual NV hours** **(hr)**
	1. GB 50,736–2012 2. GB 50,352–2019			**Climate data**	CSWD	1. Wind tunnel 2. Computational fluid dynamics (CFD) 3. Wind-ventilation rate	1. The calculation formula for the wind-ventilation potential is established 2. The NV potentials of main cities in China are estimated	5 (31)	Harbin (1929) Beijing (2884) Shanghai (3402) Kunming (3427) Guangzhou (4777)
	**Outdoor threshold**	**Outdoor dry-bulb temperature**	1. 18 °C < , & < 28 °C 2. Adaptive thermal comfort model	**Configuration data**	1. The building model with a length scale of 1:100 and dimensions of 0.2*0.2*0.16 m (depth*width*height) 2. A single 1 m*1 m window on each façade				
		**Humidity**	None						
		**Airflow**	None	**Ventilation strategy**	None				
^30^	**Scenarios & Criteria**			**Climate & Configuration data**		**Simulator/Core Methodology**	**Evaluation purpose/Highlight**	**Climate zone (Cities)**	**Annual NV hours** **(hr)**
	1. Ventilation, Thermal Comfort and Indoor Air Quality 2018, Crawford Wright Head of Design (BB 101) 2. GB 50,189–2015			**Climate data**	CSWD	1. Energy performance calculator (EPC) 2. EN ISO 13,790	1. Normative calculation approach that can reduce the impact of parametric uncertainties 2. Six scenarios in 100 cities under five standards are analyzed and displayed on index map, including underdeveloped regions	5 (100)	Harbin (1476) Beijing (1714) Shanghai (1071) Kunming (2437) Guangzhou (1285)
	**Outdoor threshold**	**Outdoor dry-bulb temperature**	Below upper threshold of adaptive thermal comfort model and higher than 12.8℃	**Configuration data**	1. GB 50,189–2010 2. GB 50,352–2005 3. GB/T 50,033–2013 4. GB 50,189–2015 5. GB 50,736–2012 6. GB 50,176–93				
		**Humidity**	70%, 80% RH						
		**Airflow**	15 ACH, 20 ACH	**Ventilation strategy**	1. Hybrid ventilation 2. Pure natural ventilation				

One of the predictable reasons is that, unlike the United States, there is no reference building (baseline) with a set of base assumptions and provisions as described in Appendix G of the ASHRAE 90.1 standard^42^. A self-built building model is tested for specific research objectives. This is, by the way, another reason why the EPC approach is chosen, which uses a different philosophy from the ASHRAE 90.1. This EPC method is normative. Based on this, each proposal is calculated and compared to a reference value (typically per square meter) predetermined over considerable functionally equivalent buildings. In this respect, neither modeling nor modeler bias is introduced. Input values for modeling are fully defined and directly related to observable regulations.

More broadly, the current climate classification is argued to no longer meet the energy design requirements with decades of global warming. This increase is indicated to be larger in China according to the 3rd National Assessment Report on Climate Change of China^35^. It is noted that there are no clear separations of the climatic characteristics of the Yangtze River Basin and the Yunnan-Guizhou Plateau in the current ASHRAE, as well as the distinction of the Tibetan Plateau from other plateaus in China. To address this concern, a revision has been proposed by refreshed climatic zone definition studies. A recent suggestion is that the boundaries between the Severe Cold zone and Cold zone as well as the Cold zone and Hot Summer and Cold Winter zone should be shifted toward the north from where they were located according to the standard published in 1993^43^.

Another reason that would aggravate the potential deviation is related to the rapid economic growth and urbanization in China. In the past two decades, with gradual awareness of the negative social pressures, the introduction of principles for building energy efficiency, and the establishment and utilization of related legal systems, further policy progress for buildings came into force continuously at the national level.

Natural ventilation is an extremely sensitive energy efficiency conservation strategy, and adequate climatic classification in terms of construction is a fundamental and essential step toward building potential estimation. Therefore, this paper is a part of a much wider applied study, which (1) investigates the optimization of the available parameters (building basic geometries, envelope properties, internal gains, and HVAC systems) in the DPM phase for full climate zones in China and then, (2) with a number of samples improvement in diverse climate zones, how various parameters and how different design options (mainly envelope properties and indoor temperature set point schedules of HVAC systems) could impact the NV potential are evaluated and optimized.

## Summary

In summary, this study is directed towards the following three aspects: (1) Using the evaluation of natural ventilation potential, which is sensitive to built environment information, as an entry point, this study explores the degree of bias caused by critical influencing parameters and inconsistencies or biases in model information during the DPM phase. This aims to advance research in this direction, suggesting that the degree of bias caused by building model information as it is transmitted to the building design application end should be known and controllable. Furthermore, the study delves deeper into investigating the advantages of probabilistic thinking in building performance simulation. (2) Through case studies in China, based on thousands of statistical tests on sensitivity parameters of natural ventilation potential under the Energy Performance Calculation (EPC) model, the study explores the fluctuation patterns of natural ventilation potential and identifies whether significant potential improvements can be achieved by optimizing key parameter relationships. This research advances the evaluation of the potential of natural ventilation (NV) cooling effectiveness in this specific direction. (3) These experimental data and natural ventilation evaluation results provide a data foundation for new approaches to develop a climate classification for building energy efficiency addressing Chinese climate characteristics.

## Material and methods

This study mainly follows sensitivity analysis. By calculating the change range of the NV potential evaluation index caused by the change in one or more uncertain factors, the influence of the change in each factor on the realization of the expected cooling effectiveness is analyzed. The main experimental steps are as follows:

1. Select uncertain factors through current related building specifications and regulations and set their range of variation.
2. Determine NV potential indicators for sensitivity analysis.
3. Calculate the influence degree of uncertain factors on the analysis indicators. Rank the influence degree of factors according to the weight.
4. Sort optimization of the factors using weights. Determine the best combination form in turn. The principle is that the factors with significant weight are determined first. Then, the best value with a smaller weight is determined. Finally, the optimization of buildings in specific climate locations is obtained.
5. Calculate the NV potential using the building model with parameter optimization. Then, the study results are displayed in map form.

Notably, in the background of being responsible for the growing concerns of climate-responsive effective building design in China, a series of policies and standards regarding thermal climate zones and their applications have been published by the government since the 1980s^44^. GB 50,189–2015^45^ is regarded as the refreshed specification in which building thermal design limit values for five climate zones are specified. In this standard, the thermal design code for building envelopes is regulated according to each thermal climate zone. The first step in involving this standard is to identify the climatic zone in which the building is located and then find the corresponding building energy efficiency limits in the standard. In this sense, building energy-efficiency limits for a specific site may vary with the climate zone at that location due to changes in the boundaries of climate zones. Hence, in this paper, the code stipulated in the GB stand series was used to limit the value range first. Then, according to GB 50,189–2015's 11 subdivisions, the value range was further concentrated through sensitivity analysis experiments. Finally, optimization could be recommended for each detailed subclimate zone. When there are trade-off options for study details that the GB series cannot explain, refer to ISO 52016^22^.

The following is a further description of data sources, building modeling, evaluation criteria, and calculation logic.

### Climate data

China’s vast territory (the land area is 9.6 million km^2^) and the diversity of terrain make its climate diverse (Fig. 11). From south to north, it spans multiple temperature zones of hot, warm, and cold, as well as a Qinghai-Tibet alpine-cold region. There is not only a year-round no-summer region in the northern part of Heilongjiang Province but also a region with four distinct seasons in the Huang-Huai River and a long-summer no-winter region on Hainan Island. From southeast to northeast, it spans humid, semihumid, semiarid, and arid regions, and different temperature zones and dry and wet regions interweave with each other. The diversity of climate characteristics leads to the diversity of building energy-saving design requirements. The hourly Chinese Standard Weather Data (CSWD) were developed by the National Meteorological Administration and Tsinghua University^30^.

### Building prototype

#### Basic geometry

As discussed in the Introduction section, there is no national reference in China for building modeling, and an office building prototype is employed, which is filtered by a study from 106 building design cases collected from the architectural design data set^32,46^. No significant vertical or lateral airflow between or across floors is observed in this template for office buildings, making it possible to limit the assessment to one typical floor, with a height of 3.6 m and window-to-wall ratio of 28% in the original, in the middle section of the building.

#### Applications

Major envelope characteristic modeling is in compliance with GB 50,189–2015 and GB50176-2016^45^. The internal gains and air conditioning parameters (HVAC activity schedules, etc.) are according to GB50034-2013^47^, GB50736-2012^48^ and GB/T50785-2012^49^. Characteristic supplements for special climate zones (such as hot summer and cold winter) are according to JGJ26-2010^50^, JGJ134-2010^51^ and JGJ75-2012^52^. Further details regarding the selected office building prototype are provided in Table 2.

**Table 2:** Envelope properties of prototypical buildings in different climate zones.

Climate zone	Sample size		Envelope design					Internal design		
			Window to wall (S&W)	Window design		Appurtenance	Envelope Heat Capacity (J/K)	Internal heat gain	Building temperature set-point schedule	Relative humility
				Window U value [W/m^2^/K]	SHGC	Shading reduction factor				
1 Severe cold	Area A	6	0.10–0.40	2.3	/	0.0–1.0	VH/H	Low/Average/High	21–30 °C	70–80 RH
			0.40–0.60	1.4						
	Area B		0.60–0.90	1.2						
	Area C	1	0.10–0.40	2.4	/					
			0.40–0.60	1.5						
			0.60–0.90	1.3						
2 Cold	Area A	12	0.10–0.40	2.5	0.52	0.0–1.0	VH/H/M	Low/Average/High	21–30 °C	70–80 RH
			0.40–0.70	1.7	0.4					
	Area B		0.70–0.90	0.3	0.3					
3 Hot Summer and Cold Winter (HSCW)	Area A	7	0.10–0.40	3	0.44	0.0–1.0	VH/H/M/L	Low/Average/High	21–30 °C	70–80 RH
			0.40–0.70	2.2	0.35					
	Area B		0.70–0.90	1.8	0.24					
4 Hot Summer and Warm Winter (HSWW)	Area A	5	0.10–0.40	4	0.44	0.0–1.0	VH/H/M/L/VL	Low/Average/High	21–30 °C	70–80 RH
			0.40–0.70	2.5	0.26					
	Area B		0.70–0.90	2	0.18					
5 Moderate	Area A	2	0.10–0.40	4	0.44	0.0–1.0	VH/H/M/L/VL	Low/Average/High	21–30 °C	70–80 RH
			0.40–0.70	2.5	0.35					
	Area B	1	0.70–0.90	2	0.24					

*Envelope heat capacity (J/K) Class^22^.

Very heavy (VH): 370,000 * Af, Heavy (H): 260,000 * Af, Medium (M): 165,000 * Af, Light (L): 110,000 * Af, Very Light (VL): 80,000 *Af.

*The shading factor is the shaded fraction of the PV field with respect to the full sensitive area for a given sun orientation (values 0 = no shade, 1 = fully shaded).

#### Set point schedules

One of the pitfalls in the HVAC activity schedules specified by a series of GB standards is that no ventilation alternative strategy is considered, especially because no NV cooling scenarios are accounted for in unoccupied periods, regardless of the fact that occupants in China have been identified to be accustomed to using natural ventilation to regulate the indoor environment with the prevalent phenomenon of operable exterior windows. Hence, in this study, the indoor temperature set point schedule is redesigned based on the schedule in GB 50,189–2015. The weekday night (0:00–7:00, 18:00–24:00, the gray part in Table 3 and full-time in the weekend direct NV cooling utilization is considered. The target set point determines whether the ambient air is favorable for indoor cooling and is also considered a main parameter influencing the NV potential. Hence, to optimize the suitable system operational mechanism, this parameter is also tested in this study. Values of set points in the schedule during nonworking hours are simplified to one as a representation. Each value was tested individually to find the most appropriate one. Values in the range of 21–30 °C were tested.

**Table 3:** Main operating schedule of the temperature control system (Test numeric range (°C)).

Building temperature set-point schedule					Office schedule						
Hour	WD_Tset_heat	WE_Tset_heat	WD_Tset_cool	WE_Tset_cool	Hour	Occ_WD	Occ_WE	App_WD	App_WE	Light_WD	Light_WE
0–1	5	5	21–30	21–30	0–1	0.1	0.1	0.1	0.1	0.1	0.1
1–2	5	5	21–30	21–30	1–2	0.1	0.1	0.1	0.1	0.1	0.1
2–3	5	5	21–30	21–30	2–3	0.1	0.1	0.1	0.1	0.1	0.1
3–4	5	5	21–30	21–30	3–4	0.1	0.1	0.1	0.1	0.1	0.1
4–5	5	5	21–30	21–30	4–5	0.1	0.1	0.1	0.1	0.1	0.1
5–6	12	5	21–30	21–30	5–6	0.1	0.1	0.1	0.1	0.1	0.1
6–7	18	5	21–30	21–30	6–7	0.1	0.1	0.1	0.1	0.1	0.1
7–8	20	5	21–27	21–30	7–8	0.5	0.1	0.5	0.1	0.5	0.1
8–9	20	5	21–27	21–30	8–9	0.95	0.1	0.95	0.1	0.95	0.1
9–10	20	5	21–27	21–30	9–10	0.95	0.1	0.95	0.1	0.95	0.1
10–11	20	5	21–27	21–30	10–11	0.95	0.1	0.95	0.1	0.95	0.1
11–12	20	5	21–27	21–30	11–12	0.8	0.1	0.5	0.1	0.8	0.1
12–13	20	5	21–27	21–30	12–13	0.8	0.1	0.5	0.1	0.8	0.1
13–14	20	5	21–27	21–30	13–14	0.95	0.1	0.95	0.1	0.95	0.1
14–15	20	5	21–27	21–30	14–15	0.95	0.1	0.95	0.1	0.95	0.1
15–16	20	5	21–27	21–30	15–16	0.95	0.1	0.95	0.1	0.95	0.1
16–17	20	5	21–27	21–30	16–17	0.95	0.1	0.95	0.1	0.95	0.1
17–18	20	5	21–27	21–30	17–18	0.3	0.1	0.3	0.1	0.3	0.1
18–19	18	5	21–30	21–30	18–19	0.3	0.1	0.3	0.1	0.3	0.1
19–20	12	5	21–30	21–30	19–20	0.1	0.1	0.1	0.1	0.1	0.1
20–21	5	5	21–30	21–30	20–21	0.1	0.1	0.1	0.1	0.1	0.1
21–22	5	5	21–30	21–30	21–22	0.1	0.1	0.1	0.1	0.1	0.1
22–23	5	5	21–30	21–30	22–23	0.1	0.1	0.1	0.1	0.1	0.1
23–24	5	5	21–30	21–30	23–24	0.1	0.1	0.1	0.1	0.1	0.1

## Calculation

### Scenarios and criteria

#### Natural ventilation potential related

Two criteria were defined to evaluate the NV cooling effectiveness.

(1) The rate of NV cooling available hours $\left({R}_{hour}\right)$) is defined as:

1 $$R_{hour} = \frac{{H_{nv} }}{{H_{ac} }}$$

where ${\text{H}}_{\text{nv}}$ (h) is the annual hours when the ambient air can meet indoor cooling needs adequately; outside these hours, ${\text{H}}_{\text{ac}}$ (h) is introduced to define those in which the cooling needs cannot be fully satisfied. ${\text{H}}_{\text{nv}}$ is defined as:

2 $$H_{nv} = {\text{IF}}({\text{AND}}\left( {\Phi C, need, ac{ } < 0, DBT{ } < \theta air, ac, RH{ } < RHT} \right),1,0$$

where *Φ*C,need, ac (W/m2) is the actual cooling need calculated before NV strategy implementation. DBT (°C) is the dry bulb temperature. θair, ac (°C) is the actual indoor temperature. RH (%) is the relative humidity. RHT (%) is the relative humidity threshold for ambient cooling. It is an upper limit custom constant.

For each hour, the calculation of the internal cooling need, *Φ*C, need, is performed once. *Φ*C, need, ac is defined as:

3 $$\Phi C,need, ac=\text{MIN}\left(0, 10*\frac{\theta air, set,cool -\theta air,0}{\theta air,10 -\theta air,0}\right)$$

where θair, set,cool is the target air temperature for a certain thermal zone, i.e., the setpoint in the activity HAVC scheduler. θair,0 is the air temperature in free-floating conditions. θair,10 is the temperature of the air obtained at a heating or cooling power of 10 W/m2.

(2)The rate of NV efficiency, i.e., cooling load reduction (${\text{R}}_{\text{cooling}}$), is defined as:

4 $$R_{cooling} = \frac{\Phi C,need, ac - \Phi C,need,nv}{{\Phi C,need, ac}}$$

where $\Phi {\text{C,need,nv}}$ (W/m2) is the cooling need calculated after NV alleviation. *Φ*C,need, nv is defined as:

5 $$\Phi C,need,{\text{ }}nv{\text{ }} = {\text{ IF}}\left( {H_{{nv}} = 0,\Phi C,need,{\text{ }}ac,0} \right)$$

(3)The ratio of the optimal value to the raw value of a certain location (Rh, Rc) is defined as:

6 $$Rh = \left( { R^{\prime}_{hour} - {\text{R}}_{{{\text{hour}}}} } \right)/R_{hour}$$

7 $$(Rc = \left( {R^{\prime}_{{{\text{cooling}}}} - R_{{{\text{cooling}}}} } \right)/R_{{{\text{cooling}}}}$$

We adjust the key performance indicators to the combination that is most suitable for the natural ventilation potential value, allowed by the regional building climate zoning rules (GB 50,189–2015). Then, by observing the ratio of the adjusted NV potential ($R^{\prime}_{hour}$, $R^{\prime}_{cooling}$) to the raw NV potential ($R_{hour}$, $R_{cooling}$), we can predict the optimal value of NV potential when the building regulations are inclined toward NV potential in a certain area.

#### Correlation related

In regression analysis, selecting the appropriate fitting method is crucial for obtaining accurate results. This study examined various regression techniques and ultimately chose linear regression based on the characteristics of the data set. The reason for choosing linear regression is that in this study, the variables such as WtoW(Window to Wall), ShadingRF(Shading Reduction Factor), Envelope Heat Capacity and so on, are largely independent of each other, and there exists a linear relationship between the independent variables and the dependent variable in the predictions. Linear regression is quick and straightforward, and it can correctly fit the strong relationships between the independent and dependent variables, allowing us to determine the weight of each variable based on the coefficients. Given the linear nature of these relationships, as well as the goal of model simplification and computational efficiency, linear regression provides the best balance between model complexity and interpretability. The linear regression model used in this study is expressed as follows:

8 $$y = \beta_{0} + \beta_{1} x_{1} + \beta_{2} x_{2} + \beta_{3} x_{3} + \ldots + \beta_{7} x_{7} + \varepsilon$$

${\text{x}}_{1}\text{, }{\text{x}}_{2}\text{, }{\text{x}}_{3}\text{, }{\text{x}}_{4}\text{, }{\text{x}}_{5}\text{, }{\text{x}}_{6}\text{, }{\text{x}}_{7}$ represent the data of WtoW (Window to Wall), ShadingRF F(Shading Reduction Factor), Envelope Heat Capacity, Internal Heat Gain, Setpoint-core, Setpoint-ambience and RH(Relative Humidity). $\beta_{1} , \beta_{2} , \beta_{3} , \beta_{4} , \beta_{5} , \beta_{6} and \beta_{7}$ are the corresponding weights for ${\text{x}}_{1}\text{,}{\text{ x}}_{2}\text{,}{\text{ x}}_{3}\text{,}{\text{ x}}_{4}\text{,}{\text{ x}}_{5}\text{,}{\text{ x}}_{6}\text{ and }{\text{x}}_{7},$ $\beta_{0}$ is the intercept, and $\varepsilon$ is the noise. At the same time, to verify the influence of the relationship between different variables on the final result, the study also adopts polynomial regression.

9 $$y = \beta_{0} + \beta_{1} x_{1} + \ldots + \beta_{7} x_{7} + + \beta_{8} x_{1}^{2} + \beta_{9} x_{1} x_{2} + \ldots + \beta_{14} x_{1} x_{7} + \beta_{15} x_{2}^{2} + \ldots + \beta_{35} x_{7}^{2} + \varepsilon$$

The scales of each value are totally different, which makes the contour of the loss function too flat, so the original data are not suitable for subsequent data processing, and the calculation process is extremely time-consuming. Therefore, converting the data from its original scale into standard deviation units should be considered to exclude the effect of scale differences among variables. During the experiment, Z Score standardization was adopted for converting the data:

10 $$\begin{array}{*{20}c} {\sigma = \sqrt {\frac{1}{N}\mathop \sum \nolimits_{1}^{N} (x_{i} - \mu )^{2} } } \\ {z_{i} = \frac{{x_{i} - \mu }}{\sigma }} \\ \end{array}$$

In this formula, ${\text{x}}_{\text{i}}$ is the original data, $\mu$ is the mean of metadata, and $\sigma$ is the standard deviation.

#### Normative assessment approach

**Figure 3:**
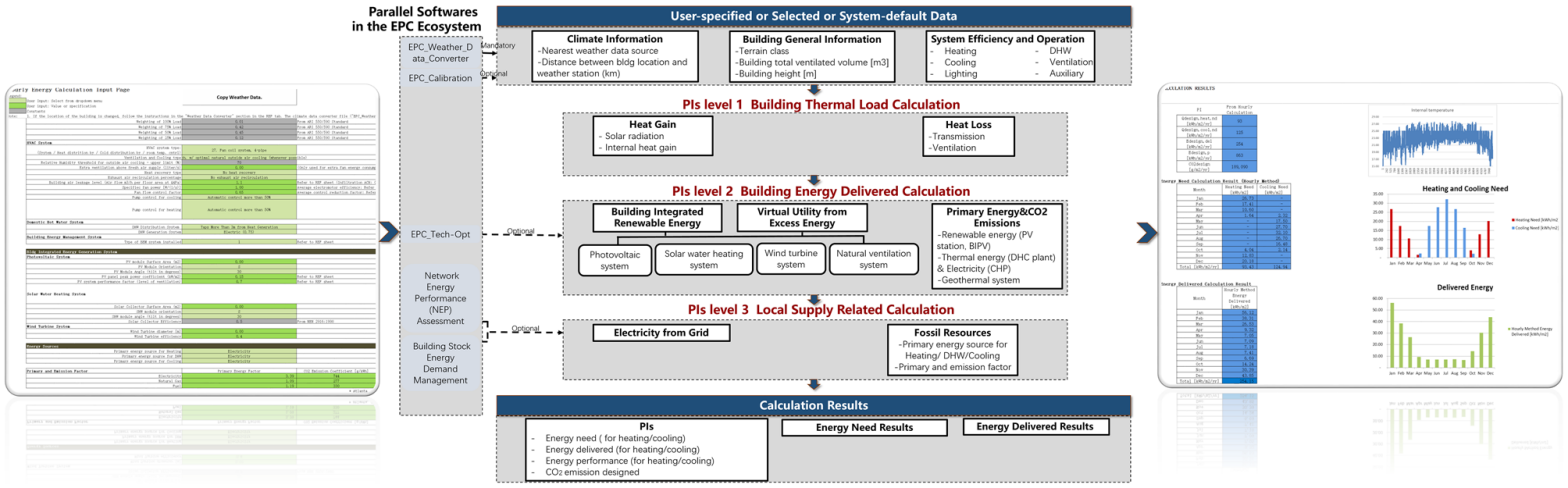
Performance indicator calculation flowchart of the normative energy performance calculator.

This paper employs the EPC calculator, which presents normative method advantages that are well suited for metric studies during the DPM phase. The calculation of EPC can be considered an extension of the performance indicator (PI) calculation, as shown in Fig. 3. Normalized indicators (PIs) are actually a measurement method defined by simplified yet indicative (virtual) experiments. The simplification of the experiment allows the derived states and aggregates of its output to be represented as closed equations that can be aggregated. These indicators can be calculated directly from relevant building and operational parameters. The resulting series of normalized calculations represent the best of both: They embody the normative properties and ease of use of physics-based simulation methods as well as rating methods.

First, a set of normative modeling assumptions ensures transparency throughout the approach, effectively eliminating modeler bias. Second, the use of normative usage scenarios enables the focus on building behavior under hypothetical conditions^53^. In level 1, 'thermal energy need' is computed in three aspects and is convenient for estimating energy performance in architectural design, even without detailed system information. These aspects entail (1) solar, internal, and system-sourced heat gains, (2) energy transmission and ventilation losses, and (3) thermal inertia. Level 2 captures 'delivered energy', or the system energy consumption designed to satisfy the energy demand from level 1. This level permits the consideration of more detailed system information, such as district heating and cooling plant options from a mix of local and distributed systems and delivered energy losses via water or air delivery. Additionally, an NV strategy is taken into account as an alternative option for HVAC systems. Level 3, on the other hand, enables the monitoring of generation and emission efficiency on the local mix utility level, including detailed primary energy and carbon emission calculations provided for the energy supply utilities and network.

## Results

### Sensitivity analysis results

For the most significant uncertainty identification from the multiple impacts of the built environment, envelope thermal capacities, internal gains, ventilation regimes, etc., as discussed in Sects. 1.3 and 1.4, and for the computational burden further reduction, a regression was applied before the hybrid potential evaluation. Two criteria, the annual rate of NV cooling suitable hour (${\text{R}}_{\text{hour}}$) and the rate of NV cooling efficiency (${\text{R}}_{\text{cooling}}$), were selected for each uncertainty run of 35 representative cities as our response variable, and all uncertain parameters were regarded as independent variables.

**Figure 4:**
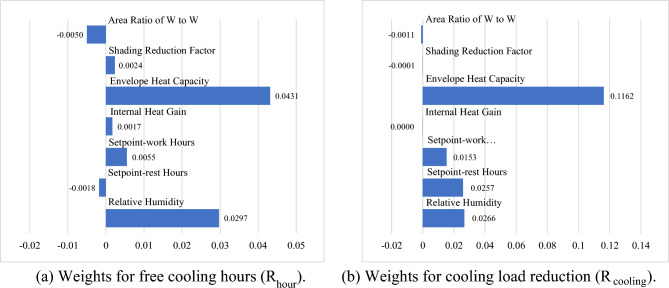
Sensitivity analysis results.

**Figure 5:**
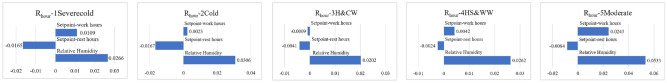
System and operation uncertainty Weights results for free cooling hours in five climate zones.

**Figure 6:**
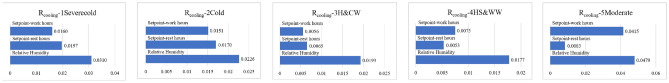
System and operation uncertainty Weights results for cooling load reduction in five climate zones.

**Figure 7:**
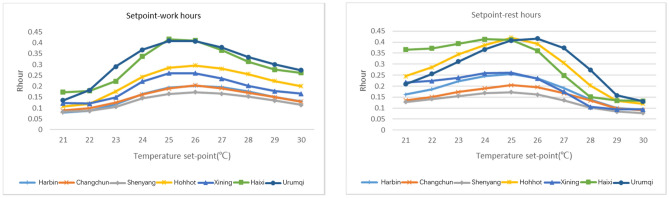
The rate of free cooling hours with respect to temperature set points in Severe Cold climate zone.

**Figure 8:**
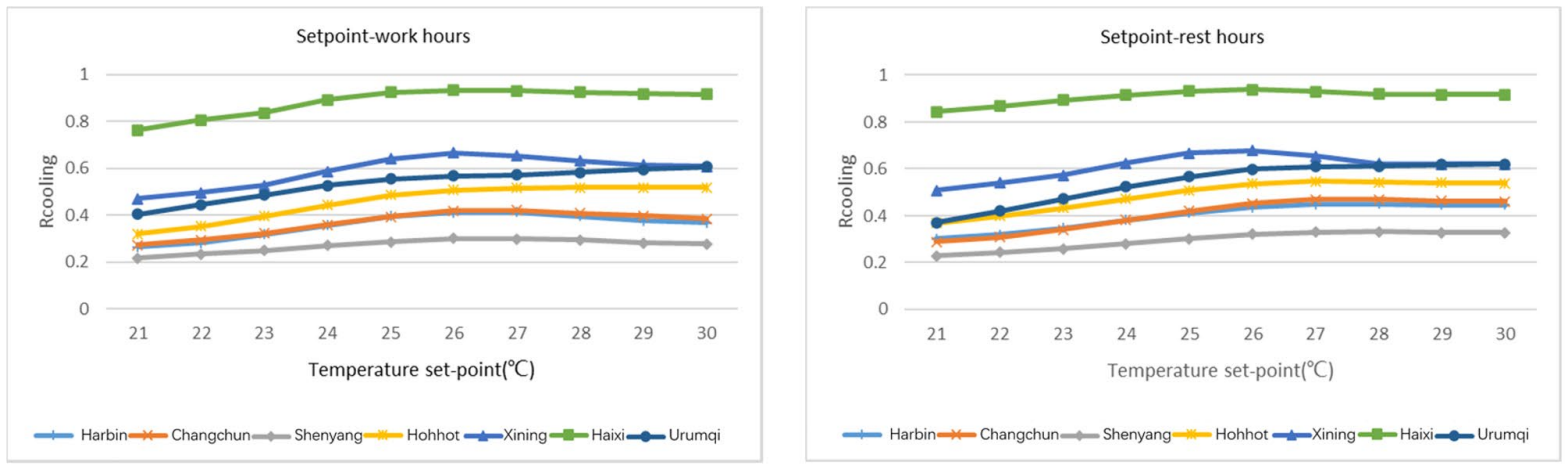
The rate of cooling load reduction with respect to temperature set points in Severe Cold climate zone.

**Figure 9:**
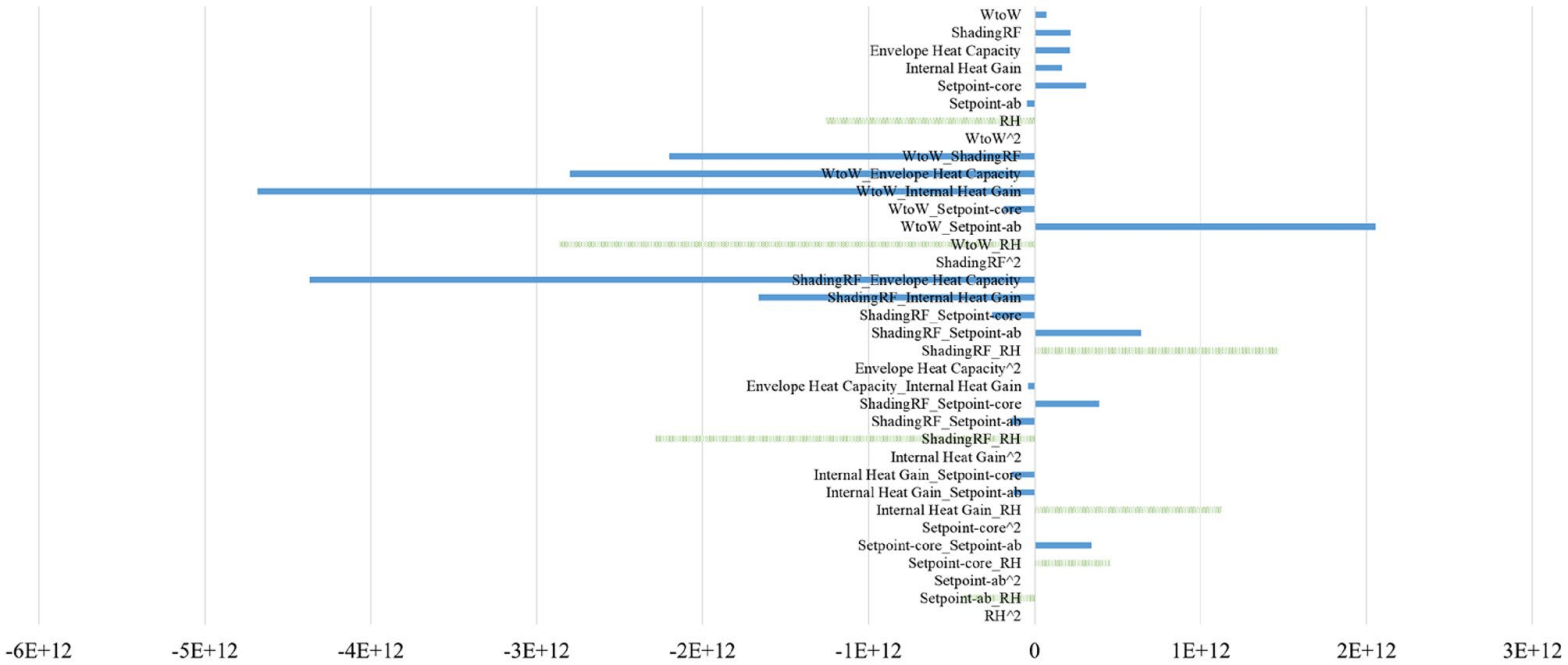
Weights (polynomial regression) for cooling load reduction (${\text{R}}_{\text{cooling}}$) in Cold climate zone.

**Figure 10:**
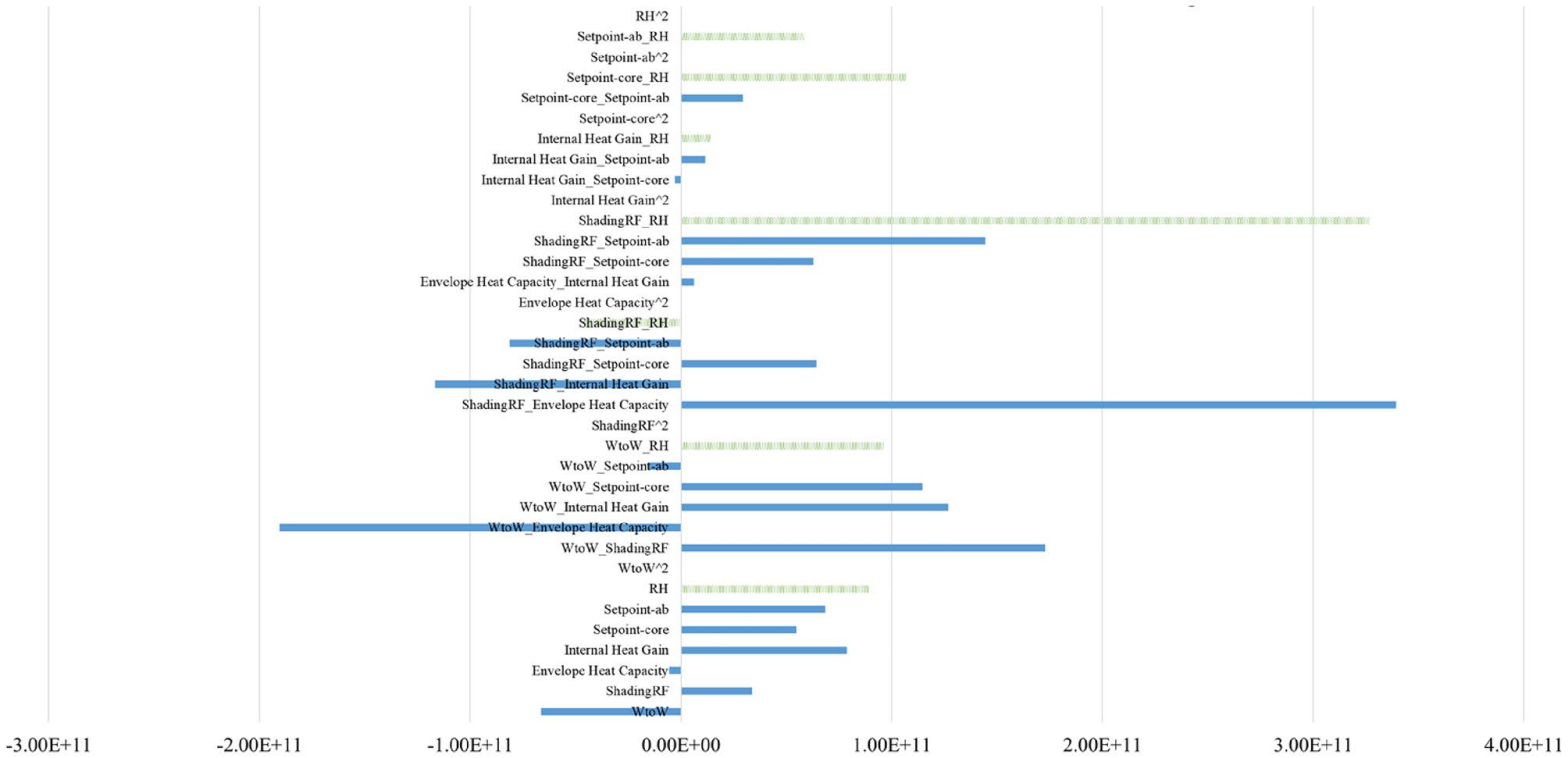
Weights (polynomial regression) for cooling load reduction (${\text{R}}_{\text{cooling}}$) in HSCW climate zone.

The sensitivity results of 35 cities in all five climate zones in China are shown in Fig. 4, where the x-axis represents the importance score, and the y-axis lists the parameter names. For both the NV suitable hours and cooling efficiency, the envelope heat capacity uncertainty dominated the sensitivity, followed by RH, which introduced crucial uncertainty. The results show that the envelope heat capacity and RH effect could lead to an approximately 7.28% (2.97%, 4.31% discretely) shift in the mean annual percentage of NV hours and a 14.28% (11.62%, 2.66% discretely) shift in the mean annual percentage of NV cooling efficiency. The second-class influencing uncertainties are regarded as ventilation regimes, represented by system and operation uncertainty (HVAC system set point schedules) in this study. The obvious inconsistent presentation of set points in the schedule during working hours (0.55% in ${\text{R}}_{\text{hour}}$, 15.25% in ${\text{R}}_{\text{cooling}}$ discretely) and nonworking hours (0.18% in ${\text{R}}_{\text{hour}}$, 25.74% in ${\text{R}}_{\text{cooling}}$ discretely) is observed, and to scrutinize the importance of these two uncertainties (working hour and nonworking hour set points), uncertainty analyses for each climate zone were executed. The results are shown in Figs. 5 and 6, with detailed explanations in Figs. 7 and 8. For the annual natural ventilation available hour potential, the uncertainty introduced by work-hour set points was negatively correlated in the midlatitude climate zone (Hot Summer and Cold Winter) in China. As the climate gradually changes to the two poles, i.e., colder or hotter, the parameter tends to show a positive correlation, and the impact gradually increases. The most uncertainty-vulnerable area is the moderate climate zone, followed by the Severe Cold climate zone. The uncertainty introduced by rest-hour set points shows negative correlations in all climate zones and was identified as a significant parameter that can be highly reflected in cold areas (north of the Yangtze River). For the annual cooling load reduction potential, the uncertainty introduced by both work-hour set points and rest-hour set points was negatively correlated in all climate zones with similar influence levels. The sensitivity of the parameter decreases gradually with increasing latitude from south to north. This overall trend is scrutinized with a multiple nonlinear regression equation, and RH introduces this significant uncertainty, as shown in Figs. 9 and 10. Figure 9 shows that only 16.61% of the uncertainties are related to RH in the cold climate zone. In the HSCW climate zone, the NV potential is more sensitive to RH, with threefold average increases (44.69%, Fig. 10).

In addition, taking Severe Cold climate zones with obvious variation in different influencing parameters as an example, we have demonstrated in detail the variation pattern of ${\text{R}}_{\text{hour}}$ and ${\text{R}}_{\text{cooling}}$ with the setting of setpoints to further explain the above presentation of positive and negative correlations (Figs. 7, 8). The reason for potential variation of the workhour setpoint parameter is obvious, that is, it is mainly the result of the three-state coupling relationship between indoor and ambient temperature difference and temperature setting. The significance of Rest is mainly to precool the place before the start of working hours. Therefore, if the setting value in the rest hour is too much higher than the setting value during working hours, it will not reach the significance of precooling. In contrast, if the rest-hour setting value is too low, outdoor air is not allowed to enter the indoor space most of the time, which will also lead to the underembodiment of potential. ${\text{R}}_{\text{cooling}}$ is much more intuitive and consistent in this regard. From this perspective, we believe cooling load reduction can better describe NV's contribution to energy conservation and emission reduction. The significance of the existence of ${\text{R}}_{\text{hour}}$ is more in terms of adjusting the static environment and improving subhealth. Consequently, we emphasize that the estimation of NV potential should not be reduced to the point of not being able to investigate nightly natural ventilation, which would be too imprecise, especially for areas with large diurnal temperature differences, which are generally deemed to have effective NV potential.

In summary, the uncertainties will be applied in future investigations of ventilation potential, including the best combination form of envelope heat capacity uncertainty, RH uncertainty, system and operation uncertainty. The area ratio of window to wall uncertainty, shading reduction factor uncertainty and internal heat gain uncertainty were neglected in the subsequent analysis.

### Natural ventilation potential estimation results

One hundred cities have been analysed, with a particular focus on those located in the Qinghai-Tibet Plateau, Yunnan-Kweichow Plateau, and the eastern region of the Inner Mongolian Plateau, and those recommended to be reassigned into new thermal zones by Lujian Bai et al. to further investigate the impact of these critical yet limited variables during the scheme design stage on NVP as a response to definition research of new thermal climate zones for building energy efficiency.

**Figure 11:**
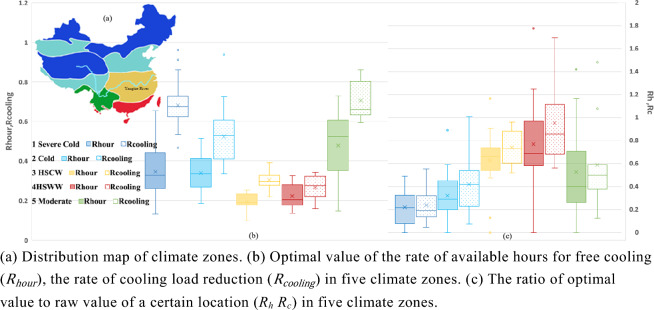
NV cooling potential with box plot at five climate zones.

Figure 11b showcases the optimized natural ventilation potential values for the five climate zones, with color-coded boxes corresponding to the color scheme of the climate zones depicted in Fig. 11a. Additionally, Fig. 11c outlines the subsequent growth rate of natural ventilation potential after optimization, explicitly differentiating between the solid-filled boxes, which signify an increase in natural ventilation hours, and dot-filled boxes, which indicate an increase in natural cooling potential.

Figure 11b shows that despite relaxing the RH to 80%, the HSCW and HSWW climate zones display relatively low natural ventilation potential values. This suggests that natural ventilation is not a favorable choice for these regions due to the underlying climatic limitations, regardless of any modifications in building design schemes. However, these regions, particularly the Yangtze River Delta, are among the most densely populated and economically developed areas in China. As a result, it would be worthwhile to assess natural ventilation benefits on a per square meter building area basis and explore any potential benefits. Moreover, considering that Shanghai, a substantial economic hub in East Asia, was subject to COVID-19 lockdowns from April to June 2022, this study demonstrates a 62% increase in natural ventilation hours and a 60% increase in cooling capacity. It is worth noting, however, that despite these positive outcomes, the potential values themselves are not sizable (${\text{R}}_{\text{hour}}$ 0.139, ${\text{R}}_{\text{cooling}}$ 0.222).

In Fig. 11, it is evident that the Severe Cold and Cold regions display favorable natural ventilation potential values comparable to the Moderate region, which has long been favored for this aspect. The Severe Cold region is characterized by strong cooling capacity, and certain areas such as Haixi (${\text{R}}_{\text{cooling}}$ 0.961, Rc 0.046) and Qamdo (${\text{R}}_{\text{cooling}}$ 0.912, Rc 0.252) in the Qinghai-Tibet Plateau demonstrate the upper limit threshold of natural ventilation potential for this region due to large diurnal temperature differences. Lhasa in the Qinghai-Tibet Plateau (${\text{R}}_{\text{hour}}$ 0.338, ${\text{R}}_{\text{cooling}}$ 0.937, Rc 0.076) has the highest natural ventilation potential value in the Cold region, while Leshan in the Sichuan Basin (${\text{R}}_{\text{cooling}}$ 0.333, Rc 1.010) displays a significant increase in optimized potential values.

Moving on, the Moderate region impressively displays natural ventilation potential values in both ventilation duration and cooling effect (Fig. 11b). Additionally, this potential has not saturated with condition restrictions, and the natural ventilation potential of this region still shows a significant increase when sensitive factors are actively addressed (Fig. 11c). Therefore, prioritizing this region for further research on natural ventilation building design schemes is advisable. The city with the best natural ventilation hours is Mengzi (${\text{R}}_{\text{hour}}$ 0.729), while the city with the best natural ventilation cooling efficiency is Deqen (${\text{R}}_{{{\text{cooling}}}}$ 0.861). Kunming, the economic core city of this climate zone and a representative city of the Yunnan-Kweichow Plateau, holds a leading position in terms of duration, cooling efficiency, and optimization improvement space.

**Figure 12:**
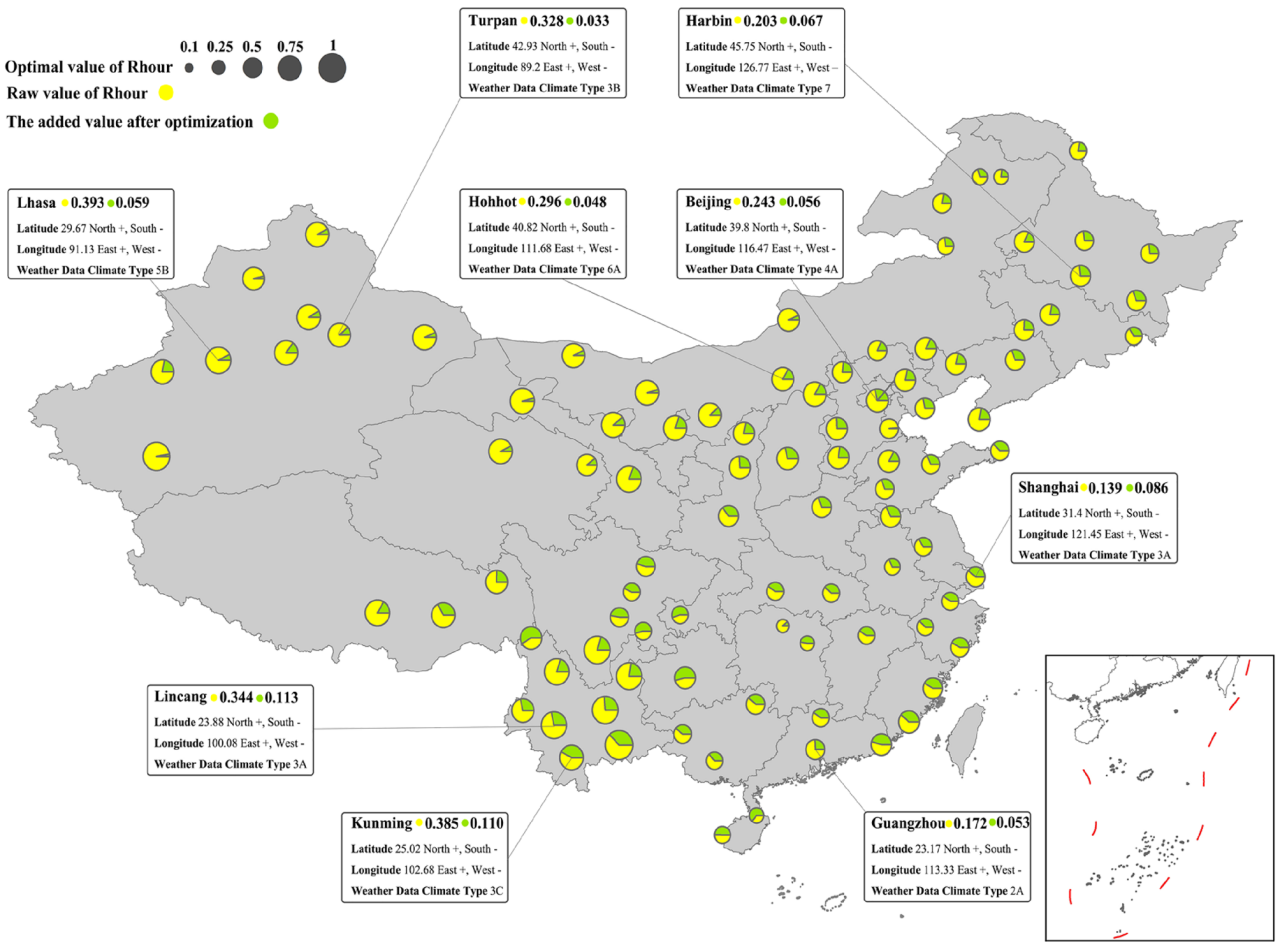
NV hours affected by the critical parameters in China.

**Figure 13:**
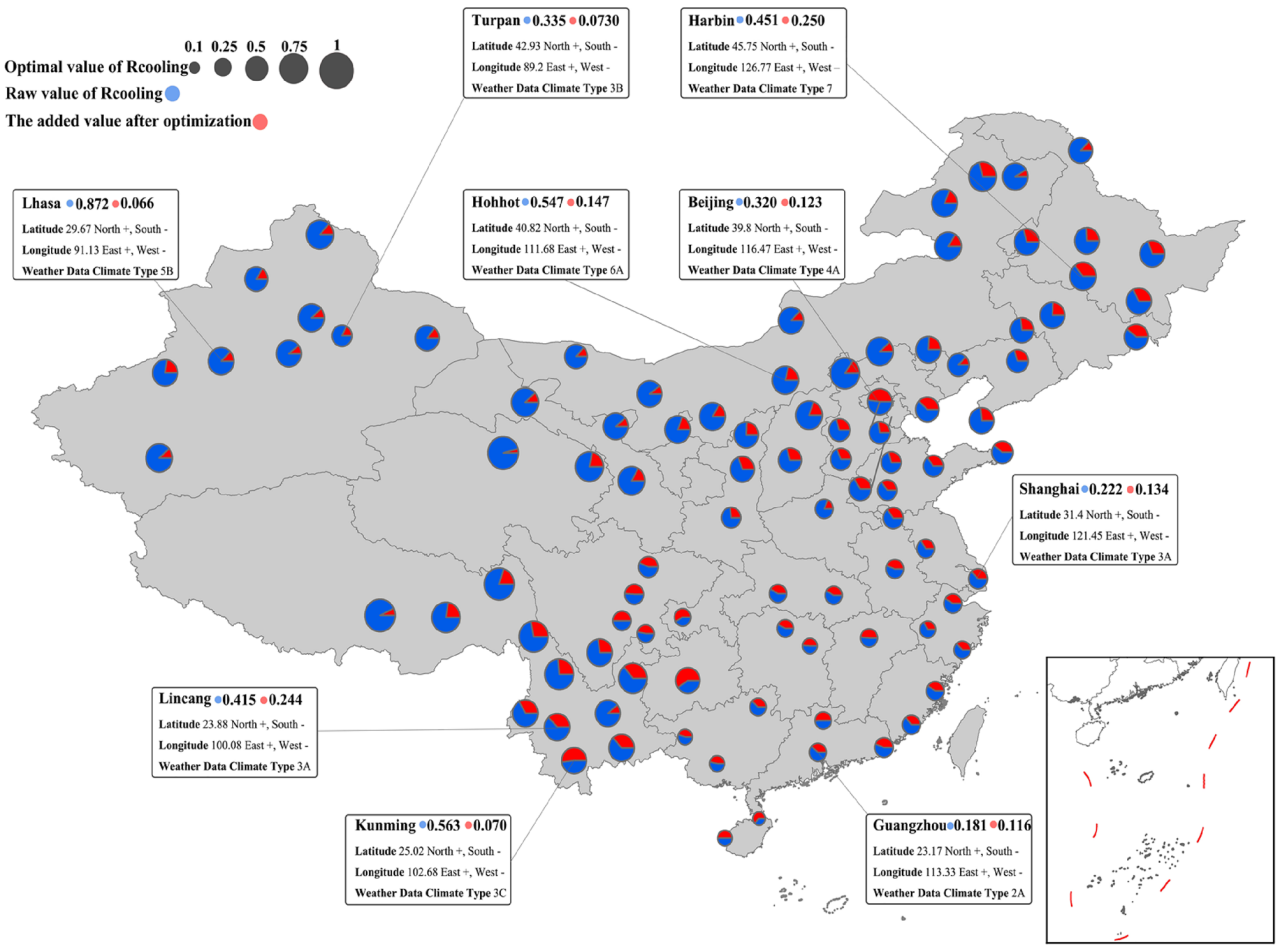
NV cooling effeteness affected by the critical parameters in China.

Figure 12 and Fig. 13 compare the specific city potentials. First, due to climatic conditions, cities in the Qinghai-Tibet Plateau and the eastern region of the Inner Mongolian Plateau, which were expected to have high potential values, did not show significant improvement in potential despite their original potential values being relatively high. Cities located in the Beijing-Tianjin-Hebei urban agglomeration, such as Dalian (${\text{R}}_{\text{cooling}}$ 0.607, Rc 0.363) and Tianjin (${\text{R}}_{\text{cooling}}$ 0.506, Rc 0.516), display good comprehensive improvement, which is a positive finding, as this region is the most economically developed area in northern China, with a cold winter and a hot summer, relying on thermal power supply near the coal-producing province of Shanxi. In-depth research on key influencing factors could contribute to setting indoor temperature elasticity and natural ventilation building design.

Second, we observe that the Sichuan Basin region is highly sensitive to influencing factors, with all tested cities displaying growth rates exceeding 50%. Further verification of natural ventilation related to this region carries meaning for economically representative cities such as Chengdu (belonging to the HSCW area, Rh 0.743, Rc 0.959) and Chongqing (belonging to the HSWW area, Rh 1.246, Rc 1.417), including design practice and climate zone research. As discussed in the introduction, China's building climate zone division has been criticized for being too rough due to its large geographical span and topographic diversity. Our study on the sensitivity of natural ventilation potential can provide some basic data for this direction.

## Discussion

The observation perspective of this study starts with a question. From a retrospective review, we found that the potential evaluation of natural ventilation (NV) cooling effectiveness in the same category based on system scales, i.e., similar meteorological uncertainty, research objectives and objects, shows significant differences, which even affect the potential ranking of representative cities of climate zones. Uncertainties added and uncertainty propagation (both model form uncertainties and parameter uncertainties) could result in large discrepancies between simulation outcomes and real scenarios, especially in the DPM phase, since in this conceptual design stage, only a few parameters are available and therefore decisive. It is necessary to review and identify the key performance indicators and explore the extent to which deviations are caused by inconsistencies or biases in model information. As a basis for more concrete research, we propose statistical tests based on quantitative evaluations in transient states to explore the rule of natural ventilation potential volatility and identify whether there is a significant potential improvement resulting from the critical parameter enhancement with the optimal relationship.

The impact of climate on building energy efficiency is complex. Research indicates that NV potential is highly sensitive to thermal mass, and the threshold of thermal mass settings is ultimately determined by the building’s climate zoning. Essentially, due to the existence of some debatable gray climate zones within the research field—areas that are classified under one climate zone in some studies and under another in other studies. The evaluation of natural ventilation potential, as a downstream data input, becomes constrained and even confusing. Notably, these areas do not just include vast uninhabited regions with difficult-to-obtain data, but also densely populated provinces like Henan, economically developed provinces like Jiangsu, provinces with abundant natural ventilation resources like Yunnan, and regions with significant diurnal temperature variations conducive to natural ventilation like Tibet. This also underscores the importance of considering multiple factors when establishing climate zoning for building energy efficiency design. These factors include not only comprehensive regional climate characteristics and the dispersion of relevant meteorological parameters but also the combined effects of climate, thermal properties of buildings, internal gains, ventilation curves, and institutional factors.

For the specific area of natural ventilation potential evaluation, given the paucity of studies on the impact of climatic dynamics on passive design, improved climate zones metrics should enhance indoor thermal comfort and energy efficiency. For example, the definition of air temperature range directly influences night ventilation, which is a crucial component of the cooling potential of natural ventilation. This aspect is currently overlooked in China’s green building guidelines. At the very least, it is advisable to consider pre-cooling indoor residual heat by ventilating for two hours before the start of the working hours.

It is indicated that defining smaller and more uniform climate zones can assist policymakers and building designers in enhancing building energy efficiency and improving indoor thermal environments. For example, a stratified climate zoning approach has been proposed, utilizing climate data (such as degree-days, relative humidity, solar radiation, and wind speed) and Hierarchical Agglomerative Clustering (HAC) based on Ward’s method. Climate zoning serves as a foundational step in formulating energy efficiency building regulations in many countries. In China, with a vast land area of 9.6 million km2, ranging from 21°N to 54°N and 74°E to 135°E, the Ministry of Housing and Urban–Rural Development has established national thermal design standards for civil buildings. China is divided into five climatic zones based on these standards. In the United States, climate zones at the national level are categorized into 19 zones according to thermal conditions (ranging from 0 to 8) and moisture conditions (moist, dry, and marine) by state. Additionally, the California Energy Commission has established 16 climate zones to provide more detailed guidance, primarily based on the average summer and winter temperatures from 600 weather stations.In Australia, the National House Energy Rating Scheme (NatHERS) divides the country’s eight climate zones into 69 sub-zones. The permissible energy loads and energy performance ratings vary across these climate zones, facilitating building comparisons under the diverse Australian weather conditions.

The work demonstrates how the simplified method can be used to generate detailed indoor operative temperature data based on various building conditions and control profiles, which are utilized to assess the cooling potential of natural ventilation at the strategic design stage. Furthermore, we can discuss the application scope and credibility of probabilistic thinking in the design phase of buildings. Introducing quasi-steady standardized calculation methods, a “normative” calculation procedure, into the strategic design research field helps address the limitations of the core algorithms in this study. Specifically, in recent years, building design tends to follow a data-driven model. This primarily involves using artificial intelligence technologies (such as Convolutional Neural Networks and Generative Adversarial Networks) to learn from computationally intensive numerical simulation cases (i.e., data sets preset for machine learning). This allows for the generation of large amounts of multidimensional parameters that align with the goals within a certain timeframe, leading to iterative optimization. This mimics the human brain’s thought process during the design phase under limited building environment information. The numerical simulations are mainly based on detailed physical models like EnergyPlus. The core algorithms of these models are not designed for the data-driven generative design settings, which feature limited quantifiable information and massive computational loads. As a result, they show limitations in balancing the accuracy of simulation results with reasonable computational loads: a. The design phase is a process where conditional information continuously inputs, increasing graphical information and decreasing abstraction. The quantities, shapes, and qualities of space are interdependent and constantly changing, leading to limited quantifiable building information. In this phase, it is necessary to make many assumptions about key parameters to conduct Design Performance Modeling (DPM). Therefore, there is a possibility of computational deviation due to insufficient assumptions. b. The detailed physical model itself also requires the introduction of assumptions and simplifications, which have been proven to similarly increase computational deviations. Currently, surrogate models are commonly used to balance computational load issues, but this further exacerbates computation result deviations. This computational process has certain time and resource costs, and along with the possible need for surrogate models, it makes the computational process nearly non-traceable. The quasi-steady standardized calculation method, while ensuring accuracy equivalent to that of detailed physical models, frees up significant computational resources. Its standardized calculation process provides transparency, which is beneficial for retrospective checks. Its programming simplicity and rapid computation make it better suited to integrate with data-driven models, embedding in the intelligent generative design workflow for buildings.

## Conclusion

This paper thoroughly investigates the potential of hybrid ventilation in various regions of China while considering a range of uncertainties, such as meteorological, building and operational uncertainties. Initially, a general overview of the potential for hybrid ventilation in China was conducted, followed by a detailed analysis to determine the degree of influence of uncertain factors on the analysis indicators in this paper. Subsequently, a sensitivity analysis was performed to eliminate unnecessary uncertainties. Finally, statistical tests were proposed based on quantitative evaluations to verify the volatility of natural ventilation potential and to identify any significant potential improvements resulting from critical parameter enhancements. The improvement in the NV energy saving percentage achieved by optimizing critical parameters resulted in an impressive increase despite the possibly higher thermal comfort risks associated with it.

Thirty-five cities under two standards are analyzed, including hybrid ventilation with a night-purge ventilation strategy. Uncertainty investigations in the DPM phase need not be performed by a full dynamic simulation. However, this is time consuming and defeats the purpose of a rapid repetitive evaluation. A full simulation would also introduce bias into the PI quantification, as no simulation can be performed without introducing assumptions and simplifications, often dependent on the type of simulation tool. The remedy against this bias is the introduction of ‘normative’ calculation procedures. These procedures are derived such that the indicators are indicative and objective indicators of a certain performance aspect. Although the resulting value cannot be taken as an absolute measure for an observable physical variable, the approach is ideal for comparative studies. An even larger advantage of a normatively declared PI is that its value can be calculated directly from the relevant set of building and operation parameters. A normative indicator is in fact a measure defined through a simplified but indicative (virtual) experiment.

In the following study, a hierarchical climatic zoning method for energy efficient building design will be applied in the Yunnan Kweichow Plateau, which is proposed as a top priority for natural ventilated building site selection in this study, considering NV potential with hierarchical agglomerative clustering (HAC) following Ward’s method. Smaller and more homogeneous climate zones could help policy-makers and building designers improve building energy efficiency while improving the indoor thermal environment. In China, unlike regions with established traditions of full air conditioning, NV is not an optional but essential choice, especially for occupants in a moderate climate. This tendency has become more distinct due to carbon peaking and carbon neutrality, i.e., the “Double Carbon Policy” of the Chinese government.

## Limitations and future work

The evaluation of natural ventilation potential is based on a quasi-steady-state standardized calculation method, rather than a full dynamic simulation. This is because full dynamic simulation is time-consuming and counterproductive for the purpose of rapid repetitive evaluation. A full simulation would also introduce biases into the quantification of performance indicators (PI), as no simulation can be performed without introducing assumptions and simplifications, which often depend on the type of simulation tool. The quasi-steady-state standardized calculation method has been introduced to the field of natural ventilation potential assessment as a remedy against this bias. The resulting values cannot be taken as absolute measures of observable physical variables; however, this approach is ideal for comparative studies. An even greater advantage of a normatively declared PI is that its value can be calculated directly from the relevant set of building and operation parameters. A normative indicator is essentially a measure defined through a simplified but indicative (virtual) experiment. The simplification of the experiment allows the derivation and aggregation of the output state of the experiment to be expressed as closed equations. The resulting set of normative calculations represents the best of both worlds: they embody the physics-based approach of simulation and the normative nature and ease of use of rating methods.

A series of energy-saving potential prediction simulations, conducted to guide architectural design, are usually performed before construction appliance activities, which typically last for 2–5 years. However, the meteorological data used for potential evaluations are based on historical average temperatures. Therefore, one of the resulting difficulties is the lag inherent in the climate data itself. Climate data, or more specifically, built environment data, are among the primary foundational data for predicting building energy consumption. However, the current trend of global warming poses challenges to the analysis and selection of climate zoning and related meteorological parameters for energy-efficient building design. In this regard, our studio has continued to explore a parallel study, which involves using reasonably predicted future meteorological data to guide architectural design schemes, and subsequently, to guide construction activities.

## Data Availability

All data included in this study are available upon request by contact with the corresponding author.

## Abbreviations

COP: Coefficient of performance of the air conditioning system
CSWD: Chinese Standard Weather Data
DBT: Dry bulb temperature
DPM: Design performance modelling
EPC: Energy performance calculator
*H*_*ac*_: Number of annual hours when the cooling need cannot be fully satisfied by outside air
*H*_*nv*_: Number of annual hours when the cooling need can be fully satisfied by outside air
HSCW: Hot Summer and Cold Winter
HSWW: Hot Summer and Warm Winter
HV: Hybrid ventilation
HVAC: Heating, ventilation and air conditioning
MHV: Machinal hybrid ventilation
NHV: Natural hybrid ventilation
NV: Natural ventilation
PDPH: Pressure difference pascal hours
PI: Performance indicator (PI)
PNV: Pure natural ventilation
*R*_*c*_: Ratio of optimal value to raw value (*R*_*cooling*_) of a certain location
*R*_*cooling*_: Rate of cooling load reduction
*R**′*_*cooling*_: Rate of cooling load reduction (adjusted)
RH: Relative humidity
*R*_*h*_: Ratio of optimal value to raw value (*R*_*hour*_) of a certain location
*R*_*hour*_: Rate of natural ventilation cooling available hours
*R**′*_*hour*_: Rate of natural ventilation cooling available hours (adjusted)
*RHT*: Relative humidity threshold for ambient cooling
ShadingRF: Shading Reduction Factor
Setpoint-ab: Setpoint-ambience
*ΦC, need, ac*: Actual cooling need calculated before the natural ventilation strategy implementation
Φ*C, need, nv*: Cooling need calculated after natural ventilation alleviation
*θair, 0*: Air temperature in free floating conditions
*θair, 10*: Temperature of the air obtained heating or cooling power of 10 W/m^2^
*θair, set, cool*: Target air temperature for a certain thermal zone
W to W: Window to Wall
