# Author Correction: Natural ventilation cooling effectiveness classification for building design addressing climate characteristics

**DOI:** 10.1038/s41598-024-73832-8

**Published:** 2024-09-25

**Authors:** Wenjing Li, Xinhui Xu, Jiawei Yao, Qingchang Chen, Zhuoyang Sun, Mehdi Makvandi, Philip F. Yuan

**Affiliations:** 1 College of Architecture and Urban Planning, Tongji University, Shanghai, 200092 People’s Republic of China; 2 School of Architecture, Shanghai Institute of Technology, Shanghai, 200235 People’s Republic of China; 3 Key Laboratory of Ecology and Energy-Saving Study of Dense Habitat, Ministry of Education, Shanghai, 200092 People’s Republic of China; 4 College of Civil Engineering, Zhejiang University of Technology, Hangzhou, 310058 People’s Republic of China

Correction to: *Scientific Reports*https://doi.org/10.1038/s41598-024-66684-9, Published on 13 July 2024

In the original version of this Article Xinhui Xu, Jiawei Yao, Mehdi Makvandi and Philip F. Yuan were incorrectly affiliated with ‘School of Architecture, Shanghai Institute of Technology, Shanghai 200235, People’s Republic of China.’ Their correct affiliations are listed below.

**Xinhui Xu, Jiawei Yao, Mehdi Makvandi and Philip F. Yuan**:

College of Architecture and Urban Planning, Tongji University, Shanghai 200092, People’s Republic of China.

**Jiawei Yao**:

Key Laboratory of Ecology and Energy-Saving Study of Dense Habitat, Ministry of Education, Shanghai 200092, People’s Republic of China.

Furthermore, Qingchang Chen was incorrectly affiliated with ‘College of Architecture and Urban Planning, Tongji University, Shanghai 200092, People’s Republic of China.’ Their correct affiliation is listed below.

School of Architecture, Shanghai Institute of Technology, Shanghai 200235, People’s Republic of China.

The original Article has been corrected.

